# Prevalence and factors associated with mental health problems among adolescents living with HIV as screened by youth peers in rural Uganda: A cross-sectional study

**DOI:** 10.1371/journal.pgph.0005289

**Published:** 2026-04-29

**Authors:** Mary Abwola Olwedo, Simon Eleku, Emmanuel Ayikobua Tiyo, Richard Mpango, Alex Imalingat, Fred Kirya, Derrick Amone, Hellen Akurut, Nelson Bunani

**Affiliations:** 1 Soroti University School of Health Sciences, Soroti, Uganda; 2 Makerere University School of Public Health, Kampala, Uganda; 3 Faculty of Medicine, Gulu University, Gulu, Uganda; NYU Grossman School of Medicine: New York University School of Medicine, UNITED STATES OF AMERICA

## Abstract

Adolescents living with HIV face numerous psychosocial challenges that increase their vulnerability to mental health problems. However, limited evidence exists on the prevalence and contributing factors among this population in the Teso region of Uganda. This study assessed the prevalence of mental health problems and associated factors among adolescents living with HIV in the Teso region. A cross-sectional study was conducted among adolescents aged 10–19 years receiving HIV care in selected high-volume health facilities in the Teso region. Data were collected using the Home, Education/Employment, Activities, Drugs, Sexuality, Suicide/Depression psychosocial assessment tool (HEADSS tool), uploaded onto Kobo Collect for digital data collection. The data were exported to Microsoft Excel, cleaned, and analyzed using STATA version 17. Descriptive statistics were used to summarize sociodemographic characteristics and estimate the prevalence of mental health problems. Logistic regression analysis was used to identify factors associated with mental health problems. The prevalence of mental health problems was 35.2%, with suicidal tendencies being the most common (31%). Factors significantly associated with mental health problems included staying with one parent (AOR = 0.71; 95% CI: 0.55-0.92; p = 0.001) and not working (AOR = 4.4; 95% CI: 1.66–11.62; p = 0.03). Mental health problems are prevalent among adolescents living with HIV in the Teso region. Supportive living arrangements were protective, while employment was associated with increased risk. Peer-led screening can aid early identification, emphasizing the need to integrate mental health services into adolescent HIV care, strengthen family and caregiver support.

## Introduction

HIV remains a major global public health challenge, with an estimated 39.9 million people living with HIV worldwide at the end of 2023, including approximately 1.5 million adolescents aged 10–19 years [1]. Similarly, in Uganda, an estimated 1.4 million people are living with HIV, including approximately 170,000 adolescents and young adults aged 15–24 years [2,3]. In 2022, the World Health Organisation (WHO) estimated that almost half of mental health disorders in adulthood start by age 14 years, but most cases are undetected or untreated [4]. Available evidence from Uganda revealed that the prevalence of any mental disorder was 22.9% in children and 24.2% in adults [5]. This study further found that the prevalence of anxiety disorders was 14.4% in children and 20.2% in adults, and current depressive disorders were 22.2% in children and 21.2% among adults. Additionally, studies in Uganda by Ashaba, Cooper-Vince [6] and Mpango, Ssembajjwe [7] found that the prevalence of depressive disorders among children and adolescents ranged between 13.7% and 16%, while suicide was at 13.7%.

Adolescents living with HIV are vulnerable to psychosocial challenges like non-disclosure, stigma, fear, anxiety, low self-esteem, and depression due to their serostatus, which can significantly affect their treatment adherence and retention in care [2]. These issues are exacerbated by their HIV status and could significantly affect treatment adherence and retention in care. As such, the Ugandan HIV policy guidelines recommend routine screening for psychosocial issues and mental health problems at every clinic visit using the HEADSS tool [2]. The recommendation is based on its established use as a structured clinical assessment tool, but the document does not report formal measures of reliability (such as internal consistency, validity coefficients, or reliability metrics) for HEADSS among HIV-positive adolescents in Uganda. In practice, although the HEADSS tool is widely used internationally for adolescent biopsychosocial screening, evidence on its psychometric reliability in specific clinical populations like HIV-positive adolescents in Uganda is limited. Some research in other contexts has applied HEADSS or culturally adapted versions to identify psychosocial issues and risk behaviors among adolescents living with HIV, suggesting it can be useful and acceptable for systematic screening [8], but formal reliability testing in Ugandan adolescents living with HIV populations remains sparse. Efforts have been made by Uganda ministry of health to train healthcare workers in the use of the HEADSS tool, and it has been integrated into Uganda’s HIV treatment guidelines [2]. Although the tool is included in the national guidelines, its practical use in routine HIV care, particularly in rural clinics, has not been well documented. Similarly, data on the use of youth peers to conduct screening using HEADSS is limited in Uganda, which depicts a gap in practice and literature.

Data on mental health screening and the prevalence of mental health problems among adolescents living with HIV, especially in rural regions like the Teso, is limited. The implementation of the HEADSS tool remains underexplored in rural settings, and knowledge and capacity gaps among healthcare workers contribute to the underreporting of mental health issues. HIV clinics in Uganda employ youth peer workers, themselves adolescents living with HIV, to facilitate the engagement and retention of other adolescents in HIV care [2]. Over time, these lay workers have received structured training, coaching, and mentorship to enhance their ability to provide high-quality HIV services.

While youth-peer-led screening has shown effectiveness in other countries, such as Malawi [8], its use in Uganda’s rural HIV care settings has been very limited. This study assessed the prevalence and factors associated with mental health problems among adolescents living with HIV in selected high-volume health facilities in the Teso region.

## Materials and methods

### Study design and setting

This was a cross-sectional study conducted among adolescents attending HIV clinics in selected high-volume health facilities in the Teso region. The Teso Region is located in the eastern part of Uganda. It is primarily inhabited by the Iteso people. The major towns include: Soroti (the largest city), Kumi, Katakwi, Amuria, Bukedea, Ngora, Serere, Kaberamaido and Kalaki. The neighboring regions: Lango in the north, to the west are Buganda and Busoga regions, to the east and southeast are Bugisu and Bukedi regions and to the northeast, Karamoja region. It also has historical significance, especially regarding the impact of conflicts in the late 20th century. Although the Teso region has an HIV prevalence of 4.2%, Soroti District records a substantially higher prevalence of 6.7%, which surpasses the national average of 5.8% [3,9]

Three out of the nine districts that comprise the Teso region were purposively selected for inclusion in the study due to their high-volume HIV clinics, which have been operational for a longer duration than others.

The selected health facilities were Soroti Regional Referral Hospital, Katakwi Hospital and Atutur Hospital. These facilities were selected because they have between 200 and 600 HIV-positive adolescents enrolled in care and treatment, providing a sufficient sample size for the study. Additionally, the HIV clinics operate twice a week but remain open daily for the enrollment of newly diagnosed HIV-positive individuals.

Each facility has a team of youth peers who assist healthcare workers in linking HIV-positive adolescents to care for and tracking those who have missed appointments or dropped out of treatment. Before this study, the youth peers hadn’t been engaged in screening using HEADSS tool. The study was conducted from March to June 2023.

### Sampling and sample size

Using the Kish Leslie formula [10], with overall prevalence of any anxiety and depressive disorder combined 13.7% (7), a design effect of 2, and an expected response rate of 90%, the required sample size was calculated to be 404 participants. Adolescents living with HIV active in care at the selected study sites were included as they came to the facility for their clinic visits. Community screening was conducted for adolescents, who received their drug refill from the community. Two youth peers from each health facility were identified by the HIV clinic in charge and trained alongside selected health workers. This was on-site training for a duration of three days, with practical sessions for both the youth peers and health workers. The training covered psychosocial assessment, identification of mental health problems using the HEADSS tool, and documentation in the psychosocial section of the HIV care card.

### Study variables

The dependent variable was mental health problems. The mental health problems were suicidal ideation, depression, anxiety and substance abuse. These were measured using the HEADSS tool [2], extracted from the national consolidated HIV prevention and treatment guidelines. The participants were asked if they lost interest in pleasurable activities in the last two weeks and if they felt low or hopeless in the last 2 weeks; a yes to both questions indicated a participant needed further assessment for suicide and depression. For anxiety, the participant was asked if they felt nervous or anxious about anything; a yes indicated the presence of an anxiety problem. For substance abuse/alcohol – the participant was asked if they took alcohol or any other substance. A yes to the question indicated alcohol or substance abuse. The assessment was planned to take about 20–30 minutes, thereafter the adolescents would be supported to receive the remaining services, without much delay. Independent variables included age, sex, religion, presence of a biological parent, HIV disclosure of status, school attendance and home environment.

### Data collection

The study was conducted on clinic days and in the community using the HEADSS tool administered by the youth peers. The WHO recommends that for adolescents specifically, peers can be a unique and powerful source of empathic support, leading to better access to services [4]. The adolescent peers participate in tracking and linking back to care adolescents living with HIV who have not kept their clinic appointments and also community drug refill for fellow peers. In some of the HIV clinics, the youth peers offer technical support such as taking anthropometric measurements under the guidance of the technical staffs etc [2]. Caregivers of identified study participants aged 10–17 consented before assent was obtained from the participants, while those who were 18–19 years old consented to participate. The adolescents living with HIV who were 18–19 years were consented by the youth peers, who then administered the semi-structured questionnaire developed from the HEADSS tool, and the information obtained was entered into a Kobo collect tool and the psychosocial section of the HIV care cards of the adolescents. Kobo collect is a digital data collection tool that allows secure, efficient, and real-time entry of survey responses on mobile devices. It was chosen for this study because it reduces data entry errors, speeds up data management, and ensures completeness and accuracy compared to paper-based methods. The participants identified with mental health problems were accompanied to the clinician’s room for further technical assessment for mental health disorders. This process was repeated until the study sample size was achieved

### Data management

The data in the Kobo collect tool was downloaded in Microsoft Excel, cleaned, and exported into STATA version 17 for analysis. Descriptive statistics such as proportions were used to present the participants’ background characteristics. Univariate analysis was done to determine the proportion of adolescents presenting with mental health problems. Logistic regression was used in bivariate and multivariable analysis to identify factors associated with mental health problems. Associations were tested at a 95% confidence interval (CI). A p-value <0.2 was used at the bivariable stage to retain variables with potential associations for inclusion in the multivariable model. Model building was done by stepwise elimination, which involved adding the variables that qualified for multivariable level one at a time while dropping those with no significance. Unadjusted and adjusted odds ratios and their 95% confidence intervals (CIs) and p-values were reported.

## Results

### Characteristics of the study participants

The mean age of participants was 14.72 years (SD = 2.69). Of the 421 participants, 52.7% (222/421) were recruited from Soroti Regional Referral Hospital. Overall, 71.3% (300/421) were currently attending school, 72.9% (307/421) were receiving first-line ART, 53.7% (226/421) lived with both parents, and 69.8% (294/421) had disclosed their HIV status to at least one significant person (Table 1).

**Table 1:** Characteristics of the study participants.

Characteristics	Frequency (n = 421)	Percentage (%)
**Health facility**		
Soroti RRH	222	52.7
Atutur hospital	111	26.4
Katakwi hospital	88	20.9
**Religion**		
Catholic	81	19.2
Anglican	260	61.8
Moslem	24	5.7
Born again	29	6.9
Others	27	6.4
**Still in school**		
Yes	300	71.3
No	121	28.7
**ART Regimen line**		
1^st^ Line	307	72.9
2^nd^ Line	108	25.7
3^rd^ Line	6	1.4
**Whom the adolescent stays with**		
Both parents	226	53.7
Others*	195	46.3
**Disclosed HIV status**		
Yes	294	69.8
No	127	30.2
**Employment history**		
No	394	93.6
Yes	27	6.4
Sex		
Male	243	57.8
Female	178	42.3
Age category		
10-14 years	209	
Male	128	61.2
Female	81	38.8
15-19 years	212	
Male	115	54.2
Female	97	45.8

Note. *Other people the adolescent stayed with include one parent alive, siblings, grandmother, auntie, and uncle.

Among males, 52.7% (128/243) were aged 10–14 years, while among females, 54.5% (97/178) were aged 15–19 years.

In univariate analysis, 35.2% (148/421) of adolescents reported experiencing at least one mental health problem. The prevalence of mental health problems was higher among adolescents aged 15–19 years (64.2%) compared to those aged 10–14 years (35.8%) (Table 2).

**Table 2:** Prevalence of mental health problems among the study participants.

Age	N = 421 (%)	Prevalence of common mental health problems (%)
10-14 Years	209 (49.6)	35.8 (53/148)
15-19 Years	212 (50.4)	64.2 (95/148)
Total	421	35.2 (148/421)

In univariate analysis, 30% (127/421) of adolescents reported depressive symptoms, 25% (106/421) reported anxiety, 31% (132/421) reported suicidal tendencies, and 2% (7/421) reported alcohol or substance abuse (Table 3).

**Table 3:** Common mental health problems among the study participants.

Reported mental health problems	Frequency		Percentage of common mental health problems
	Yes	No	
Depression	127	294	30
Anxiety	106	315	25
Suicidal tendency	132	289	31
Alcohol/substance abuse	7	414	2

### Factors associated with mental health problems among adolescents living with HIV in Teso region

As shown in Table 4, at bivariate analysis, still in school, age group, who you stay with and employment history were associated with mental health problems. The odds of mental health problems among the age group 15–19 years were 2.4 times the odds among those aged 10–14 years (UOR = 2.4; 95% CI = 1.58-3.61; p=<0.001). Among those still in school, the odds of mental health problems were 0.29 times that of those not in school (UOR = 0.29; 95% CI = 0.18-0.46; p=<0.001). The adolescents who stay with one parent had 3.9 times the odds of mental health problems among those who stay with both parents (UOR = 3.9; 95% CI = 2.55-5.97; p=<0.001). The odds of mental health problems among the adolescents who were working were 9.5 times the odds among those who were not working (UOR-9.5; 95% CI = 3.51-25.67; p = 0.001).

**Table 4:** Factors associated with mental health problems on bivariate analysis.

Variable	Unadjusted OR	95% CI	P-Value
**Age group**			
10-14 Years	1		
15-19 Years	2.4	[1.58-3.61]	<0.001*
**Still in school**			
No	1		
Yes	0.29	[0.18-0.46]	<0.001*
**Who do you stay with**			
Both parents	1		
Other	3.9	[2.55-5.97]	<0.001*
**Disclosure**			
Yes	1		
No	0.67	[0.43-1.02]	0.075
**Are you currently employed or in the past**			
No	1		
Yes	9.51	[3.51-25.67]	0.001*

In multivariable analysis, who the adolescents stay with and adolescents in employment, remained significantly associated with mental health problems. Compared with adolescents living with both parents, those living with one parent showed significantly lower odds of mental health problems (AOR = 0.71, 95% CI: 0.55–0.9, p = 0.0.001). Among the working adolescents, the odds of mental health problems were 4.4 times the odds among those not working (AOR = 4.4; 95% CI = 1.66-11.62; p = 0.03) Table 5.

**Table 5:** Factors associated with mental health problems on multivariate analysis.

Variable	Adjusted OR	95% CI	P-Value
**Age Group**			
10-14 Years			
15-19 Years	0.81	[0.62-1.06]	0.131
**Still in school**			
No			
Yes	1.24	[0.87-1.78]	0.238
**Who do you stay with**			
Both parents			
Other	0.71	[0.55-0.92]	0.001*
**Are you currently employed or in the past**			
No			
Yes	4.4	[1.66-11.62]	0.003*

Note. * P-value is significant at ≤ 0.05, UOR-Un adjusted odds ratio, AOR- Adjusted odds ratio, CI-Confidence interval.

## Discussion

The study aimed to determine the prevalence of mental health problems and associated factors among adolescents living with HIV in the Teso region. The findings revealed that 35.2% of adolescents experienced mental health problems, with suicidal tendencies (31%) and depressive symptoms (30%) being the most prevalent. The reported prevalence of depression was notably higher than that found among adolescents and children living with HIV in Masaka and Kampala, which stood at 13.7% (7). Similarly, in South Africa, Haas AD [11] reported that 4.4% of adolescents living with HIV experienced severe depression, while 2.9% had suicidal concerns. In line with our findings, a study in Cameroon reported suicidal ideation in 36.4% of adolescents [12]. In Botswana, Merrian Brooks [13] found that 33.1% had anxiety, 44.3% had depression, and 15.0% reported symptoms of suicidality or self-harm. The high prevalence observed in our study could be attributed to the use of youth peer-led screening. Youth peers are non-technical lay workers whose capacity is developed on the job while supporting HIV clinic activities under the supervision of technical staff. They are primarily assigned to provide psychosocial support to fellow adolescents [4]; however, in some clinics, task-shifting strategies have been employed to expand their roles beyond this function The screening was conducted using the HEADSS tool, a psychosocial assessment instrument designed to identify signs and symptoms indicative of potential mental health disorders in adolescents. These findings highlight the need for national mental health policies to integrate routine mental health screening into HIV care for adolescents, strengthen adolescent mental health services at all levels of the health system, and invest in youth-friendly, peer-led psychosocial support programs.

The study found that adolescents who were currently employed or had previously worked had significantly higher odds of experiencing mental health problems. This finding appears counterintuitive, as unemployment is more commonly associated with psychological distress. However, the relationship between employment and adolescent mental health is highly context dependent. In some high-income settings, structured or part-time employment has been shown to reduce financial stress and improve psychosocial wellbeing among adolescents [14]. In contrast, in many low-resource contexts, adolescent employment often reflects underlying multidimensional poverty rather than opportunity, and poverty itself has been independently associated with poorer mental health outcomes among adolescents through pathways such as household economic strain, disrupted schooling, and cumulative psychosocial stress [15]. Moreover, adolescent work in such settings is frequently informal, physically demanding, or time-intensive, conditions that have been linked to increased psychological distress and emotional and behavioral problems [16,17]. Furthermore, because this study used a cross-sectional design, reverse causality cannot be excluded; adolescents with existing mental health difficulties may be more likely to disengage from school and enter the labour force earlier, as poor mental health has been shown to increase the risk of school dropout and subsequent transitions into non-education or work pathways later in life [18]. The findings highlight the importance of policies that support school retention, address socioeconomic pressures related to early employment, and enhance access to mental health services within adolescent HIV care programs.

The study also found that living arrangements were associated with mental health outcomes. Adolescents living with one caregiver had lower odds of mental health problems compared with those living with both parents. Although this finding may appear unexpected, it may reflect differences in household dynamics, caregiving consistency, or levels of family stress and conflict rather than the number of caregivers alone [19]. However, the cross-sectional design limits conclusions about the underlying mechanisms or direction of this relationship

More complex family dynamics may increase psychosocial stressors and vulnerability to depression, anxiety, and substance use [20]. Adolescents living with both parents may face increased responsibilities and greater psychosocial stressors, leading to higher susceptibility to mental health issues, such as depression and anxiety [21,22]. These findings highlight the importance of strengthening psychosocial support for adolescents across diverse family structures and ensuring targeted support for those experiencing unstable or high-stress household environments within HIV care and mental health programs.

### Strengths and limitations

The study utilized peer-led mental health screening using the HEADSS tool, which is crucial for the early identification of mental health issues among adolescents living with HIV. The nature of the HEADSS tool being subjective was overcome by comparing the findings with those of other studies that used standard tools such as PHQ-9, meant for diagnosis of depression. Self-reported mental health outcomes may be biased, but additional screening by technical staff helped validate the findings. Another limitation was purposive sampling method was used, which could have introduced selection bias and limiting the generalizability of the study findings. To overcome this challenge, all the adolescents at the study sites had the opportunity to participate in the study.

## Conclusion and implication

The prevalence of mental health problems among adolescents living with HIV in the Teso region was 35.2%, with depression and suicidal tendencies each affecting about 30% of participants. Supportive living arrangements were protective, while being currently or previously employed was associated with increased risk. These findings underscore the value of peer-led mental health screening in identifying at-risk adolescents and highlight the need for policies that integrate mental health services into adolescent HIV care and strengthen family and caregiver support systems

## Supporting information

S1 File HEADSS TOOL– Psychosocial assessment tool, extracted Consolidated Guidelines for Prevention and Treatment of HIV in UGANDA, 2018. (PDF)

S1 Data Data set. (XLSX)
