# Supplementary material for: Prevalence and factors associated with mental health problems among adolescents living with HIV as screened by youth peers in rural Uganda: A cross-sectional study
Source: PLOS Glob Public Health. 2026 Apr 29;6(4):e0005289. doi: 10.1371/journal.pgph.0005289 (PMC13127912; doi:10.1371/journal.pgph.0005289)
Supplement: S1 File — (PDF) [file pgph.0005289.s001.pdf]

A1

HEADSS TOOL – Psychosocial assessment tool

Service Package for Adolescents (10-19years)

Common issues	PSS Service Package
• **Peer pressure**<br>• **Non-disclosure**<br>• **Non-adherence**<br>• **Stigma and discrimination**<br>• **Fear and anxiety**<br>• **Low self esteem**<br>• **Lack of knowledge on sexuality e.g. body changes.**<br>• **Depression**<br>• **Drug use**	• Screening for PSS issues (Use HEADSS assessment tool)<br>• Life skills education (skills of knowing and living with self, skills of living with others and skills of effective decision making)<br>• Sexuality education<br>• HIV Testing Services<br>• Risk reduction measures (partner reduction, drugs and alcohol abuse, defilement, un intended pregnancies through family planning, early marriages)<br>• Counselling on healthy relationships<br>• Adherence counselling support<br>• Coping/emotional support<br>• Stigma reduction measures<br>• Referral and Linkage to peer support groups, education, nutrition support and livelihoods programmes<br>• Adolescent friendly services at health facilities<br>• Parental guidance<br>• Recreation services<br>• Disclosure (consider age)

Screening for Psychosocial Issues among Adolescents (1)

Home Education/Eating/Exercise Activities Drugs/Depression Sexuality Suicidality/Safety (HEADSS)	
**Home, situation, Family**	•Who lives with the young person? Where?<br>•Do they have their own room?<br>•What are relationships like at home?<br>•What do parent and relatives do for a living? Ever institutionalized? Incarcerated? Recent moves? Running away?<br>•New people in home environment?<br>•Have you disclosed your HIV status? If yes, with whom? If not, what are the reasons?<br>***May use the Genogram/Family tree***
**Education and employment**	School/grade performance--any recent changes? Any dramatic past changes?<br>Favourite subjects--worst subjects? (include grades)<br>Any years repeated/classes failed Suspension, termination, dropping out?<br>Future education/employment plans?<br>Any current or past employment?<br>Relations with teachers, employers--school, work attendance?

Screening for Psychosocial Issues among Adolescents (2)

Home Education/Eating/Exercise Activities Drugs/Depression Sexuality Suicidality/Safety (HEADSS)	
**Activities**	On own, with peers (what do you do for fun? where? when?)<br>With family?<br>Sports--regular exercise?<br>Church attendance, clubs, projects?<br>Hobbies--other activities?<br>Reading for fun--what?<br>TV--how much weekly--favourite shows?<br>Favourite music?<br>Does young person have car, use seat belts?<br>History of arrests--acting out--crime?
**Drugs**	Use by peers? Use by young person? (include tobacco, alcohol)<br>Use by family members? (include tobacco, alcohol)<br>Amounts, frequency, patterns of use/abuse, and car use while intoxicated?<br>Source--how paid for?

Screening for Psychosocial Issues among Adolescents (3)

Home Education/Eating/Exercise Activities Drugs/Depression Sexuality Suicidality/Safety (HEADSS)	
**Sexuality**	Experience with sex<br>Number of partners?<br>Degree and types of sexual experience and acts?<br>Masturbation? (normalize)<br>History of pregnancy/abortion?<br>Sexually transmitted diseases--knowledge and prevention?<br>Contraception? Frequency of use? Comfort with sexual activity, enjoyment/pleasure obtained?<br>History of sexual/physical abuse?
**Suicide and Depression**	Use PHQ-2 and suicide screening tool

Screening for Depression amongst PLHIV using PHQ-2

This is aimed at screening clients for possible depressive disorders. Health workers should screen all clients at every visit . Clients found to be eligible for further assessment should be referred for psychiatry services

Use: The PHQ-2 is not to establish a final diagnosis or to monitor depression severity, but rather to screen for depression as a "first-step" approach.

Scoring: If response is Y to both questions, client should be referred for mental Health evaluation

1. Has client lost interest in pleasurable activities in the last 2 weeks?

2. Has client felt low/down or hopeless in the last 2 weeks?
